# Effect of Bicuspid Versus Tricuspid Aortic Valve Morphology on the Fate of the Ascending Aorta

**DOI:** 10.1161/JAHA.124.038013

**Published:** 2025-04-10

**Authors:** Malin Granbom Koski, Michael Dismorr, Hanna M. Björck, Christian Olsson, Fredrik Bredin

**Affiliations:** ^1^ Department of Molecular Medicine and Surgery Karolinska Institutet Stockholm Sweden; ^2^ Department of Cardiothoracic Surgery Karolinska University Hospital Stockholm Sweden; ^3^ Department of Medicine Karolinska Institutet Stockholm Sweden; ^4^ Division of Cardiovascular Medicine, Center for Molecular Medicine Karolinska University Hospital Stockholm Sweden

**Keywords:** adverse aortic events, ascending aortic aneurysm, bicuspid aortic valve, Cardiovascular Surgery, Valvular Heart Disease, Aneurysm

## Abstract

**Background:**

Bicuspid aortic valves are associated with ascending aortic pathology, but their impact on long‐term outcomes, including aortic growth and adverse events, remains unclear.

**Methods and Results:**

This prospective cohort‐study included adult patients undergoing aortic valve surgery or ascending aortic surgery at a single center (2007–2013). The primary outcome was aortic diameter growth; secondary outcomes included all‐cause mortality and adverse aortic events. Inverse probability of treatment weighting was used to adjust for baseline differences. Among 570 patients, 204 underwent echocardiographic follow‐up, and 566 were followed for adverse aortic events. At 10‐year follow‐up, ascending aortic diameter increased significantly (mean 4 mm, *P*<0.001), with no difference between patients with BAV and TAV (*P*=0.68). After multivariable adjustment, there was no difference in all‐cause mortality (HR, 0.87 [95% CI, 0.65–1.18]), but BAV was associated with a decreased risk of adverse aortic events (HR, 0.39 [95% CI, 0.19–0.82]). Concomitant ascending aortic surgery was associated with an increased risk of adverse aortic events in patients with TAV (HR, 8.89 [95% CI, 3.36–23.6]) but was associated with a decreased risk in patients with BAV (HR, 0.06 [95% CI, 0.01–0.29]).

**Conclusion:**

Ten years after surgery, ascending aortic growth occurred regardless of valve morphology. Adverse aortic events were more common in patients with TAV, whereas patients with BAV benefited from concomitant ascending aortic surgery. These findings suggest a more liberal approach to ascending aortic surgery in patients with BAV undergoing valve replacement, but improved risk stratification is needed.

**Nonstandard Abbreviations and Acronyms**
BAV: bicuspid aortic valve
TAV: tricuspid aortic valve

**Clinical Perspective**

**What Is New?**

- There is a significant growth of ascending aortic diameter irrespective of aortic valve morphology, but there is no difference in aortic diameter growth between patients with tricuspid and bicuspid aortic valves after 10 years of follow‐up.
- Surgical aortic valve replacement with concomitant ascending aortic surgery is associated with a high risk of subsequent aortic adverse events in patients with tricuspid aortic valves, but concomitant aortic valve replacement in patients with bicuspid aortic valves was associated with a low risk of subsequent adverse aortic events.

**What Are the Clinical Implications?**

- Concomitant ascending aortic surgery should be generously performed in patients with bicuspid aortic valves who undergo aortic valve replacement to decrease the risk of future adverse aortic events.

Bicuspid aortic valve (BAV) is the most common congenital heart abnormality, with a prevalence of approximately 1%.^1^ Patients with BAV more frequently develop aortic valvular pathologies and have a 50% risk of requiring cardiac surgery throughout their lifetime due to valvular disease.^1^ Furthermore, patients with BAV have an increased risk of developing aortic aneurysms, a condition commonly referred to as BAV aortopathy. The pathophysiology behind BAV aortopathy is not fully understood but has been suggested to be of embryonic origin and likely involves both genetics and hemodynamic disturbances.^2^, ^3^, ^4^

According to European and American guidelines, concomitant ascending aortic surgery in patients with BAV undergoing aortic valve replacement should be considered in patients with an aortic diameter of >50 mm and >45 mm, respectively.^5^, ^6^ This prophylactic surgery on the ascending aorta is aimed at decreasing the risk of adverse aortic events, such as aortic dissection and rupture. However, as reflected by the difference in aortic diameter threshold between the American and European guidelines, sufficient knowledge to identify patients at risk for adverse aortic events is lacking. Although aortic diameter is indeed a predictor for adverse aortic events, patients with normal diameters are not exempt from risk.^7^ Studies investigating the fate of the aorta in terms of aortic diameter growth and adverse events in relation to valve morphology are still sparse and mostly consist of small cohorts with limited follow‐up. Therefore, we performed a prospective cohort study to evaluate the association between aortic valve BAV/tricuspid aortic valve (TAV) morphology and ascending aortic diameter growth, and long‐term clinical outcomes, during a follow‐up period of 10 years.

## METHODS

### Study Design

This was a single‐center prospective observational cohort study. The study was approved by the regional Human Research Ethics Committee Stockholm (approval number 2006/784‐31/1, with amendments 2014/960‐32 and 2017/1302‐32). Written informed consent was signed by all participants in accordance with the Helsinki declaration. Due to Swedish data protection laws, the data cannot be shared.

### Study Population

The study population consisted of patients who were a part of ASAP (Advanced Study of Aortic Pathology) study.^8^ The ASAP study population consists of patients who underwent aortic valve surgery or ascending aortic surgery at the Karolinska University Hospital in Stockholm, Sweden between January 17, 2007 and February 14, 2013. Inclusion criteria were age ≥18, with aortic valve disease or ascending aortic dilation without coronary disease. Exclusion criteria were other planned concomitant valve surgery, connective tissue disorders, or withdrawal from the study.

### Study Groups and Outcomes

The study groups were defined as patients having BAVs versus TAVs, as identified by the surgeon during the primary operation. Patients with unicuspid valves were categorized as BAV.

The primary outcome was change in ascending aortic diameter as measured perioperatively compared with follow‐up at 10 years. Index ascending aortic diameter measurements were made from perioperative transesophageal echocardiographic recordings of aortic long‐axis views at 120° to 140° obtained on the operating table under general anesthesia before initiation of surgery. The follow‐up aortic measurements were obtained in the parasternal long‐axis view of transthoracic echocardiography 10 years after the patient's primary surgery. Measurements were performed at end‐diastole by leading‐edge technique as recommended by the American Society of Echocardiography and the European Association of Cardiovascular Imaging.^9^ All echocardiograms were recorded by state‐of‐the‐art equipment and interpreted by 1 of 2 expert physicians specialized in clinical physiology. Patients were excluded from the ascending aortic diameter growth analysis if they died before follow‐up, the follow‐up echocardiography was not performed (patient declined or nonresponse), the follow‐up diameter measurement was missing, concomitant ascending aortic surgery was performed at the index operation, or an aortic event in the ascending aorta occurred before follow‐up. Secondary outcomes were all‐cause mortality and aortic events. Aortic events were defined as an aortic dissection, rupture or aneurysm, with or without surgical or endovascular intervention, or death from any of these causes. Additionally, we analyzed aortic events requiring a surgical or endovascular intervention separately. Aortic diameter thresholds for intervention were guided by contemporary European guidelines^10^, ^11^; however, individual decisions were reached after discussion including the aortic team and the patient.

### Data Sources

Per the ASAP study protocol, pre‐, peri‐ and postoperative health data were obtained through medical records. In addition, patients also completed questionnaires on medical history, hereditary conditions, lifestyle habits, and medications. Additional medical and surgical information, follow‐up echocardiography data, mortality, and aortic events were obtained from patient electronic medical records.

### Statistical Analysis

Baseline characteristics were described as means±SDs for continuous variables. Categorical variables were described as frequencies and percentages. A 2‐sided *P* value ≤0.05 was considered significant.

Ascending aortic diameter growth was measured as the difference between the aortic diameter at follow‐up and the perioperative aortic diameter in millimeters. The Wilcoxon rank‐sum test was used to test for significance between the study groups with BAV and TAV. The Wilcoxon signed‐rank test was used to test for statistical significance in growth within the study groups.

For the outcomes of all‐cause mortality and aortic event, time‐to‐event was defined as the time in days from the date of surgery to the date of event or the end of follow‐up on March 16, 2023, whichever came first. Crude all‐cause mortality was estimated using the Kaplan–Meier estimator and the Aalen–Johansen estimator while accounting for the competing risk of death for the aortic event outcome. Significance was estimated using the hazard ratio (HR) as obtained from a univariate flexible parametric (Royston–Parmar) survival model with 3 degrees of freedom. In the weighted analyses, a weighted univariate flexible parametric survival model with robust variance estimation was used. The weights were obtained as detailed below. We also calculated restricted mean survival time restricted at 15 years. This is a robust measure that can be interpreted as the mean survival time, calculated as the area under the Kaplan–Meier curve restricted at a specified time point.^12^ To adjust for potential confounding, we performed weighted analyses using optimization‐based treatment weights to estimate the average treatment effect.^13^ The weight model included age, preoperative hypertension, patient sex, previous inguinal hernia,^14^ and smoking status. Balance was estimated using standardized and absolute mean difference, where a difference of <0.1 was considered an acceptable balance.^15^ There were no missing data for the variables included in the weighting. As a sensitivity analysis, flexible parametric survival models together with a Markov multistate model was used to estimate the cumulative incidence of aortic events in the weighted sample while accounting for the competing risk of death. The code used in the analyses will be shared upon request to the corresponding author.

## RESULTS

**Figure 1:**
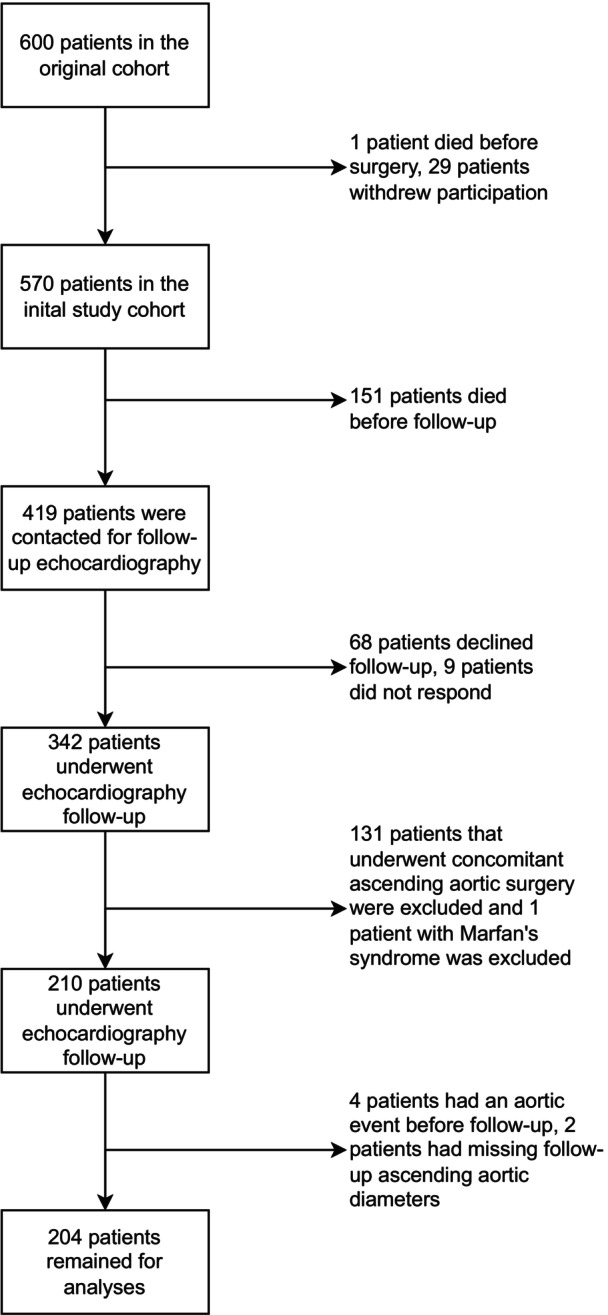
Flow chart depicting patients eligible for aortic growth analysis.

**Figure 2:**
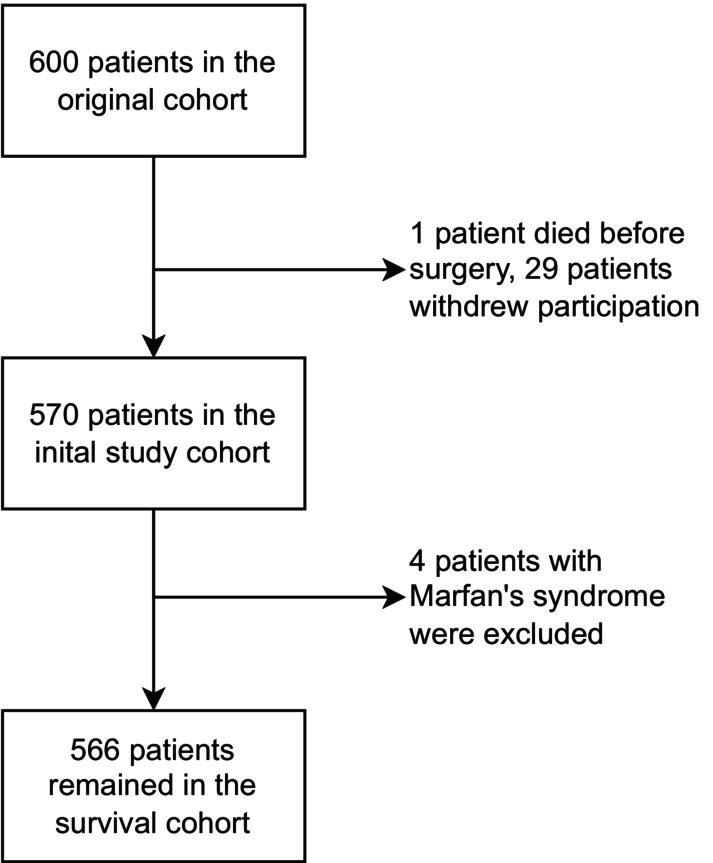
Flow chart depicting patients eligible for clinical outcomes analysis.

After exclusion, 204 of the original 600 patients were analyzed for aortic growth at 10 years. The most common reason for exclusion was death before the follow‐up echocardiography, which occurred in 151 patients. Analysis of the clinical outcomes (aortic events and survival) was performed in 566 of the original 600 patients. The most common reason for exclusion was withdrawn participation (29 patients). Flow charts illustrating the exclusion of patients for the 2 outcome groups are presented in Figures 1 and 2.

The mean age was 64 years (SD 12 years), and 32% were women. The following variables had missing data: body mass index (3.7%), valve prosthesis type (0.7%), hypertension (0.5%), prior stroke (0.5%), valve size (0.5%), prior myocardial infarction (0.4%), prior pulmonary embolism or deep vein thrombosis (0.4%), and perioperative ascending aortic diameter (0.2%). Baseline characteristics are presented in Table 1. Isolated aortic valve replacement was the most common procedure in both groups (77% and 66% for patients with TAV and BAV, respectively), followed by isolated ascending aortic surgery in patients with TAV (14%), and ascending aortic surgery with concomitant aortic valve replacement in patients with BAV (23%). The distribution of concomitant surgeries is shown in Table S1. After weighting, the absolute mean difference was <0.01 for all included variables (Figure S1).

**Table 1:** Baseline Characteristics on 566 Patients Who Underwent Aortic Valve or Ascending Aortic Valve Surgery at Karolinska University Hospital Between January 2007 and March 2013 According to Aortic Valve Morphology

Valve type	Overall	TAV	BAV	SMD	Missing
No.	566	239	327		
Age, y (mean±SD)	63.9±12.3	69.7±10.3	59.6±11.9	0.906	0.0
Female sex (%)	183 (32.3)	89 (37.2)	94 (28.7)	0.181	0.0
Body mass index, kg/m^2^ (%)				0.235	3.7
<18.5	3 (0.6)	0 (0.0)	3 (1.0)		
18.5–24.9	188 (34.5)	71 (30.7)	117 (37.3)		
25–29.9	222 (40.7)	94 (40.7)	128 (40.8)		
>30	132 (24.2)	66 (28.6)	66 (21.0)		
Smoker (%)				0.163	0.0
Current smoker	48 (8.5)	15 (6.3)	33 (10.1)		
Previous smoker	166 (29.3)	77 (32.2)	89 (27.2)		
Never smoked	352 (62.2)	147 (61.5)	205 (62.7)		
Prior atrial fibrillation (%)	68 (12.0)	39 (16.3)	29 (8.9)	0.226	0.0
Prior myocardial infarction (%)	30 (5.3)	20 (8.4)	10 (3.1)	0.229	0.4
History of cancer (%)	79 (14.0)	43 (18.0)	36 (11.0)	0.199	0.0
Chronic obstructive pulmonary disease or asthma (%)	46 (8.1)	19 (7.9)	27 (8.3)	0.011	0.0
Diabetes (%)				0.164	0.0
Type 1	5 (0.9)	1 (0.4)	4 (1.2)		
Type 2	55 (9.7)	29 (12.1)	26 (8.0)		
Not diabetic	506 (89.4)	209 (87.4)	297 (90.8)		
Hyperlipidemia (%)	94 (16.6)	49 (20.5)	45 (13.8)	0.180	0.0
Hypertension (%)	297 (52.8)	148 (62.2)	149 (45.8)	0.332	0.5
Prior stroke or transient ischemic attack (%)	54 (9.6)	36 (15.1)	18 (5.6)	0.317	0.5
Prior pulmonary emboli or deep vein thrombosis (%)	28 (5.0)	15 (6.3)	13 (4.0)	0.103	0.4
Previous inguinal hernia (%)	48 (8.5)	14 (5.9)	34 (10.4)	0.167	0.0
Perioperative ascending aortic diameter (mean±SD)	39.2±9.1	38.1±10.1	40.1±8.1	0.209	0.2
Ascending aortic surgery (%)	181 (32.0)	57 (23.8)	124 (37.9)	0.308	0.0
Aortic root surgery (%)	85 (15.0)	28 (11.7)	57 (17.4)	0.163	0.0
Valvular reconstructive surgery (%)	25 (4.4)	8 (3.3)	17 (5.2)	0.092	0.0
Aortic valve replacement surgery (%)	487 (86.0)	197 (82.4)	290 (88.7)	0.179	0.0
Valve prosthesis type (%)				0.491	0.7
Bioprosthetic aortic valve	337 (60.0)	162 (68.1)	175 (54.0)		
Mechanical valve prosthesis	146 (26.0)	34 (14.3)	112 (34.6)		
No valve replacement	79 (14.1)	42 (17.6)	37 (11.4)		
Valve size, mm (%)				0.524	0.5
18–21	148 (26.3)	87 (36.7)	61 (18.7)		
22–24	178 (31.6)	62 (26.2)	116 (35.6)		
>24	158 (28.1)	46 (19.4)	112 (34.4)		
No valve replacement	79 (14.0)	42 (17.7)	37 (11.3)		

Numbers are n (%) unless otherwise noted. BAV indicates bicuspid aortic valve; SMD, standardized mean differences; and TAV, tricuspid aortic valve.

### Aortic Growth

The mean age among patients analyzed for aortic growth was 61 years (69 years mean age in the group compared with TAV with 58 years mean age in the group with BAV). Overall, 31% were, with similar proportions in the groups with TAV and BAV. Baseline characteristics for patients analyzed for aortic growth is shown in Table S2.

**Figure 3:**
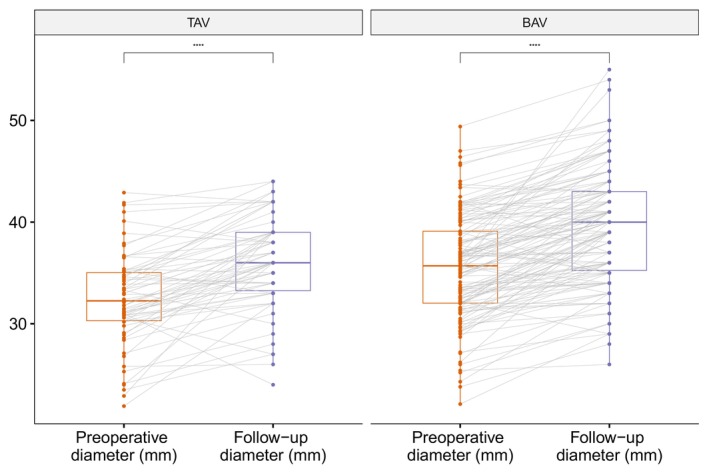
Perioperative and 10‐year follow‐up aortic ascending diameter as measured by transesophageal echocardiography, respectively. Stratified according to bicuspid or tricuspid native aortic valve morphology. ****P<0.0001, BAV indicates bicuspid aortic valve; mm, millimeter; and TAV, tricuspid aortic valve.

**Figure 4:**
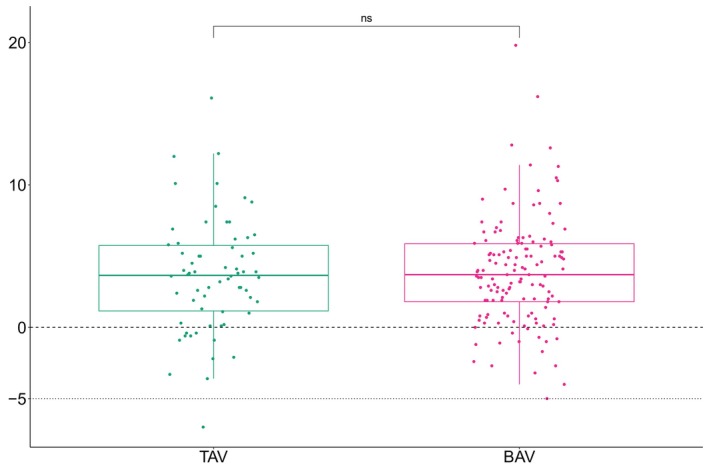
The difference in perioperative ascending aortic diameter and 10‐year follow‐up measurements in millimeter as measured by transesophageal and transthoracic echocardiography, respectively. Stratified according to bicuspid or tricuspid native aortic valve morphology. BAV indicates bicuspid aortic valve; ns, nonsignificant; and TAV, tricuspid aortic valve,

The average time between primary surgery and follow‐up echo measurement was 10.3 years. Patients with BAV had significantly larger ascending aortic diameters compared with patients with TAV, both perioperatively (mean 36 mm versus 32 mm, *P*<0.001) and at follow‐up (mean 40 mm versus 36 mm, *P*<0.001). There was a significant ascending aortic growth within both groups (*P*<0.001 and *P*<0.001 respectively) (Figure 3). There was no difference in average ascending aortic growth between patients with BAV and TAV (4 mm versus 4 mm, *P*=0.68) (Figure 4).

Ascending aortic growth and diameter growth difference according to sex are shown in Figures S2, S3. Ascending aortic diameter difference according to aortic valve morphology and pathology is shown in Figure S4.

### Survival

**Figure 5:**
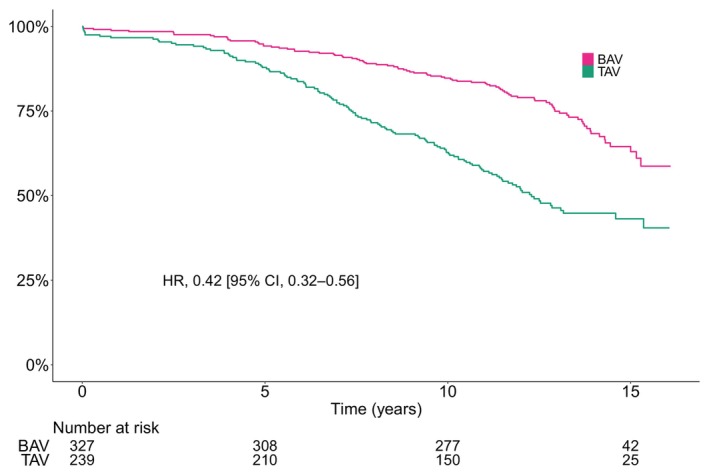
Crude Kaplan–Meier estimated survival according to bicuspid and tricuspid native aortic valve morphology. BAV indicates bicuspid aortic valve; HR, hazard ratio; and TAV, tricuspid aortic valve.

**Figure 6:**
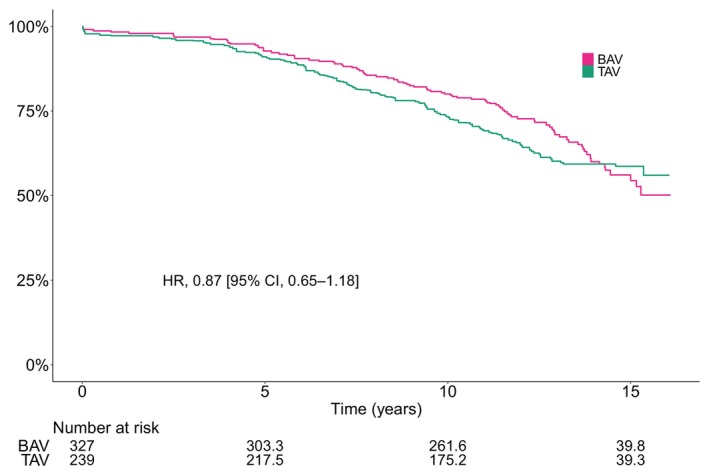
Weighting adjusted Kaplan–Meier estimated survival according to bicuspid and tricuspid native aortic valve morphology. BAV indicates bicuspid aortic valve; HR, hazard ratio; and TAV, tricuspid aortic valve.

During a mean follow‐up of 11 years (max 16 years), 216 of the 566 patients included for analysis died. In crude analysis, BAV was associated with better survival (HR, 0.42 [95% CI, 0.32–0.56]), with a restricted mean survival time of 13.1 years (95% CI, 12.7–13.4 years) compared with 11.1 years (95% CI, 10.5–11.6 years) for patients with TAV. The crude cumulative incidence of all‐cause mortality at 10 years was 37% (95% CI, 31%–43%) and 15% (95% CI, 11%–19%) for patients with TAV and BAV, respectively. The number of events per total person‐years and Kaplan–Meier estimated cumulative incidence at 1, 5, 10, and 15 years are shown in Table 2. The crude incidence rate of all‐cause mortality per 100 person‐years was 5.1 (95% CI, 4.3–6.1) and 2.3 (95% CI, 1.9–2.9) for patients with TAV and BAV, respectively. However, after age and sex adjustment, the estimated incidence rate was similar in both groups (4.1 per 100 person‐years) (Table 3). Consequently, after weighting, there was no significant difference between patients with BAV and TAV (HR, 0.87 [95% CI, 0.65–1.18]). The difference in restricted mean survival time at 15 years between BAV and TAV was 0.3 years (95% CI, –0.4 to 1 years) after weighting. The crude and weighted Kaplan–Meier curves are shown in Figures 5 and 6, respectively.

**Table 2:** Crude Cumulative Incidence and Events Per Person Years for All‐Cause Mortality, Aortic Events, and Dissection or Rupture Following Surgery at Karolinska University Hospital Between 2007 and 2013 Classified According to Tricuspid or Bicuspid Native Aortic Valve Morphology, % (95% CI)

	Events/PY	1 year	5 years	10 years	15 years
All‐cause mortality					
TAV	125/2444	3 (1–6)	12 (8–16)	37 (31–43)	57 (50–64)
BAV	91/3905	1 (0–2)	6 (3–8)	15 (11–19)	36 (29–42)
Total	216/6349	2 (1–3)	8 (6–11)	25 (21–28)	45 (39–50)
Aortic events					
TAV	21/2364	0.4 (0.0–1.2)	2.1 (0.3–3.9)	6.7 (3.5–9.9)	9.6 (5.6–13.6)
BAV	13/3800	0.6 (0.0–1.5)	1.2 (0.0–2.4)	2.4 (0.8–4.1)	4.8 (2.1–7.5)
Total	34/6164	0.5 (0.0–1.1)	1.6 (0.6–2.6)	4.2 (2.6–5.9)	6.8 (4.5–9.1)
Dissection or rupture					
TAV	8/2392	0.4 (0.0–1.2)	0.8 (0.0–2.0)	2.9 (0.8–5.1)	3.4 (1.1–5.8)
BAV	2/3856	0.0 (0.0–0.0)	0.0 (0.0–0.0)	0.3 (0.0–0.9)	0.8 (0.0–2.1)
Total	10/6248	0.2 (0.0–0.5)	0.4 (0.0–0.8)	1.4 (0.4–2.4)	1.9 (0.7–3.2)

Aortic events and dissection or rupture outcomes using Aalen–Johansen estimator accounting for the competing risk of death. BAV indicates bicuspid aortic valve; PY, person‐years; and TAV, tricuspid aortic valve.

**Table 3:** Incidence Rates Per 100 Person‐Years of All‐Cause Mortality, Aortic Events, and Dissection or Rupture Following Surgery at Karolinska University Hospital Between 2007 and 2013 Classified According to Tricuspid or Bicuspid Native Aortic Valve Morphology, % (95% CI)

	All‐cause mortality	Aortic events	Dissection or rupture
Crude			
TAV	5.1 (4.3–6.1)	0.9 (0.5–1.4)	0.3 (0.1–0.7)
BAV	2.3 (1.9–2.9)	0.3 (0.2–0.6)	0.1 (0.0–0.2)
Total	3.4 (3.0–3.9)	0.6 (0.4–0.8)	0.2 (0.1–0.3)
Age and sex adjusted			
TAV	4.1 (3.4–4.8)	0.9 (0.5–1.4)	0.4 (0.1–0.6)
BAV	4.1 (3.1–5.1)	0.4 (0.2–0.7)	0.1 (0.0–0.3)

Age‐ and sex‐adjusted incidence rates were obtained from a Poisson model. BAV indicates bicuspid aortic valve; and TAV, tricuspid aortic valve.

### Aortic Events

During a mean follow‐up 11 years (maximum 16 years), 34 of the 566 patients included for analysis had an aortic event (21 in patients with TAV, and 13 in patients with BAV). Among patients with TAV, 2 had surgery and 4 received endovascular treatment due to aneurysm, compared with 6 with aneurysm surgery and 3 with endovascular treatment in the group with BAV. Four patients had a type A aortic dissection, 3 with TAV and 1 with BAV. All patients who had a type A aortic dissection had an isolated aortic valve replacement at the index operation, except 1 patient with TAV who had a xenograft (porcine) root. The distribution of type of aortic event is shown in Table S3. The distribution of aortic event location according to aortic valve morphology is shown in Table S4. Baseline characteristics stratified according to adverse aortic events is shown in Table S5.

The crude cumulative incidence of aortic events at 10 years was 6.7% (95% CI, 3.5%–9.9%) and 2.4% (95% CI, 0.8%–4.1%) for patients with TAV and BAV, respectively. The number of total aortic events (and dissection and rupture events only) per total person‐years and Aalen–Johansen estimated cumulative incidence at 1, 5, 10, and 15 years are shown in Table 2. The crude incidence rate of aortic events per 100 person‐years was 0.9 (95% CI, 0.5–1.4) and 0.3 (95% CI, 0.2–0.6) for patients with TAV and BAV, respectively. After age and sex adjustment, the estimated incidence rate was 0.9 (95% CI, 0.5–1.4) and 0.4 (95% CI, 0.2–0.7) in patients with TAV and BAV, respectively (Table 3).

**Figure 7:**
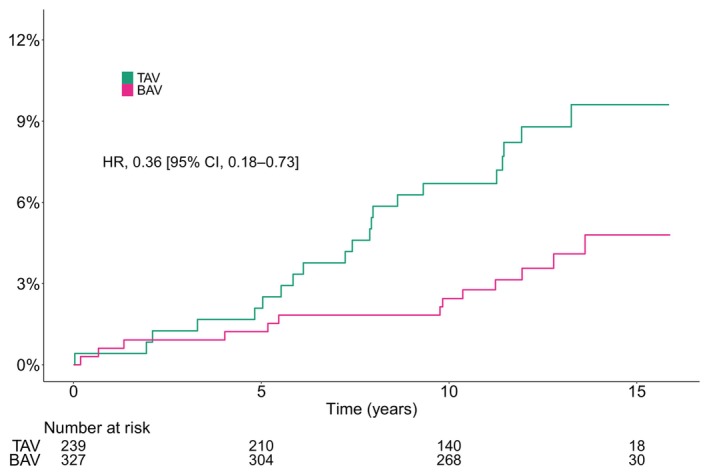
Crude Aalen–Johansen estimated cumulative incidence of aortic events according to bicuspid native aortic valve morphology. BAV indicates bicuspid aortic valve; HR, hazard ratio; and TAV, tricuspid aortic valve.

**Figure 8:**
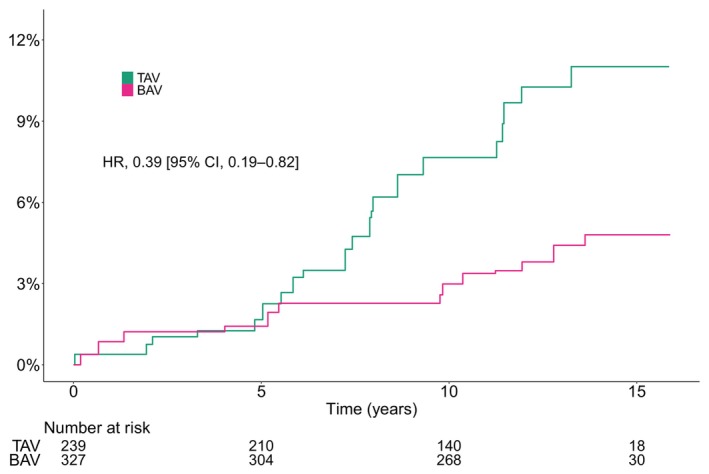
Weighting adjusted Aalen–Johansen estimated cumulative incidence of aortic events according to bicuspid native aortic valve morphology. BAV indicates bicuspid aortic valve; HR, hazard ratio; and TAV, tricuspid aortic valve.

In crude analysis, BAV was associated with a decreased risk for aortic events (HR, 0.36 [95% CI, 0.18–0.73]) (Figure 7). The significance remained after inverse probability of treatment adjusting (HR, 0.39 [95% CI, 0.19–0.82]) (Figure 8).

Concomitant ascending aortic surgery in patients with TAV was associated with an increased rate of aortic events (24.6%). Conversely, concomitant ascending aortic surgery in patients with BAV was associated with a lower rate of aortic events during follow‐up (2.4%). The crude number and proportion of aortic events per patients with BAV and TAV with and without concomitant ascending aortic surgery is shown in Table 4 and Figures S5 and S6. After multivariable adjustment, the main effect of concomitant ascending aortic surgery was associated with an increased risk of aortic events (HR, 8.89 [95% CI, 3.36–23.6]). Conversely, the HR for the interaction between BAV and concomitant ascending aortic surgery was 0.06 (95% CI, 0.01–0.29) (Table 5).

**Table 4:** Number of Patients and Aortic Events (%) Among 566 Patients

Valve morphology	Concomitant ascending aortic surgery	No.	Aortic events	Aortic events (%)
TAV	Ascending aortic replacement	57	14	24.6%
	No ascending aortic replacement	182	7	3.8%
BAV	Ascending aortic replacement	124	3	2.4%
	No ascending aortic replacement	203	10	4.9%

BAV indicates bicuspid aortic valve; and TAV, tricuspid aortic valve.

**Table 5:** Coefficients of a Cox Model for Aortic Events Including Aortic Valve Morphology, Concomitant Ascending Aortic Surgery, and Its Interactions Among 566 Patients

Variable	HR (95% CI)	*P* value
BAV vs tricuspid aortic valve (reference group)	1.41 (0.51–3.95)	0.5
Concomitant ascending aortic surgery	8.89 (3.36–23.6)	<0.001
Age	1.03 (0.99–1.07)	0.11
Female sex	0.80 (0.38–1.68)	0.6
Previous inguinal hernia	0.62 (0.18–2.11)	0.4
Smoking status		
Current smoker	…	
Previous smoker	0.86 (0.30–2.46)	0.8
Never smoked	0.41 (0.15–1.13)	0.086
BAV and ascending aortic surgery interaction	0.06 (0.01–0.29)	<0.001

BAV indicates bicuspid aortic valve; and HR, hazard ratio.

## DISCUSSION

In this prospective, observational cohort study, we found a significant growth of the ascending aorta 10 years after aortic valve replacement, irrespective of the native aortic valve morphology. However, there was no difference in aortic growth between patients with BAV and TAV morphology. There was an increase in all‐cause mortality rate in patients with TAV, but this significance disappeared after adjusting for age. TAV was associated with an increased risk for aortic events, and this association was further strengthened by concomitant ascending aortic surgery. Conversely, in patients with BAV, concomitant ascending aortic surgery was associated with a decreased risk for aortic events.

In a study conducted by Longi and colleagues,^16^ the growth rate and clinical outcomes of 431 patients with BAV, with or without root or ascending aortic aneurysms, were examined. They found that in patients with normal aortic diameters at baseline, the ascending aorta grew at a rate of 0.2 mm per year (*P*=0.01). In contrast, there was no growth of the ascending aorta among patients who had a dilated ascending aorta at baseline. In our study, we found a mean growth rate of 4 mm over 10 years, irrespective of native aortic valve morphology. This corresponds to 0.4 mm per year or double the growth rate as reported by Longi, although in absolute terms, the growth rate can be considered similar. In terms of clinical outcomes, they reported 2 reoperations for ascending aortic aneurysm during follow‐up and a 10‐year mortality rate of 2% to11%. In our study, 6 patients with BAV and 2 with TAV underwent surgery for aortic aneurysm. The 10‐year mortality was 37% and 15% for TAV and BAV, respectively. Most likely, the higher number of events and increased mortality rate observed in our study is related to the longer mean follow‐up time of 11 years compared with a mean follow‐up of 5 years in their study.

A notable finding in our study was the decreased risk of future aortic events among patients with BAV undergoing concomitant ascending aortic surgery. Thus, in this patient group, concomitant repair of the ascending aorta seems almost curative. In patients with TAV undergoing concomitant aortic surgery, on the other hand, the risk of future events in distal segments was high. It is well known that the underlying pathophysiology of aortic disease differs between patients with TAV and BAV, and we have recently suggested an embryonic origin of BAV aortopathy.^17^ The results presented here strengthen this hypothesis as an embryonic origin behind disease development would restrict aneurysm formation to the proximal part of the ascending aorta, leaving distal segments of a different embryonic origin intact. The support for embryonic involvement is also further strengthened by a recent cohort study including 25 556 newborns in Denmark, reporting an aortopathy prevalence of 33% in newborns with BAV.^18^ Moreover, the continued aortic growth following BAV repair, during which proximal aortic blood flow is restored, argue in favor of an embryonic or genetic cause.

The low event rate seen in the group with BAV also confirms previous clinical findings. For example, Zafar and colleagues showed a higher age‐adjusted event‐free survival after 10 years in patients with BAV^19^ concluding that the ascending aortic diameter threshold should not differ between patients with TAV and BAV. In a study by Itagaki et al., the risk for clinical outcomes after surgical aortic valve replacement in patients with Marfan syndrome, BAV, or controls (assumed to have TAV) was investigated, showing an increased mortality rate among controls.^20^ However, as in our study, patients with BAV tended to be younger, and after adjusting for age, the mortality rate was similar between groups. In contrast to our study, Itagaki found an association between BAV and increased risk for both new aortic aneurysms (HR, 1.93 [95% CI, 1.36–2.75]) and aortic surgery due to aneurysm or dissection (HR, 2.34 [95% CI, 1.37–4.02]) compared with controls. To what extent prophylactic ascending aortic surgery should be performed in BAV, however, needs to be further investigated. Notably, Russo^7^ recommends concomitant ascending aortic replacement in patients who undergo surgical aortic valve replacement, irrespective of aortic diameter. Although this recommendation is tempting with our results, other studies have failed to show a decreased risk of death or aortic events following generous ascending aortic replacement in patients with BAV.^21^ We believe that an improved risk stratification beyond aortic diameter will help ensure that the right treatment, at the right time, is offered.

Several studies have examined potential biomarkers that might be used in conjunction with other risk factors, including aortic diameter, sex, age, and valve morphology. Radiological modalities, such as 4‐dimensional magnetic resonance flow, has also been shown to be good at predicting the likelihood of patients developing ascending aortic aneurysms.^2^ A better risk stratification will help to identify patients with an increased risk for aortic event who would benefit from early intervention.^22^

### Strengths and Limitations

Our results are based on a large database including detailed information on valve morphology, comorbidity, and long‐term follow‐up aortic measurements for both the clinical and aortic diameter outcomes. Follow‐up related to survival is complete, and the study dropout rate was low. This provides us with a unique possibility to interrogate long‐term aortic growth in detailed patient groups and with great power.

This study also had some limitations. First, due to the observational nature of the study, residual confounding might influence the observed associations between aortic valve morphology and outcomes. However, we believe that the most important potential confounders were considered by inverse probability of treatment weighting and outcome modeling. Second, there are limitations associated with echocardiography as a modality to estimate aortic diameters. Echocardiography is known to have a high degree of interexaminer variability. This problem was mitigated by using the same 2 examiners both preoperatively and during follow‐up. However, with 10 years between examinations, some degree of intraexaminer variability is likely. Third, there is a possibility of errors during the data collection process, which is always a risk when using data from electronical medical records. However, the individuals performing the data collection reviewed the entries multiple times. No external team reviewed the collected data.

### Conclusions

In this prospective observational cohort study, we found a significant 10‐year growth of the ascending aortic diameter, irrespective of aortic valve morphology and with no difference between patients with TAV and BAV. However, TAV was associated with an increased risk of adverse aortic events compared with BAV. Importantly, concomitant ascending aortic surgery in patients with BAV had a protective effect for future aortic events and seemed almost curative. This suggests that ascending aortic surgery should be generously performed in patients with BAV who undergo surgical aortic valve replacement, although better risk stratification is needed.

## Disclosures

None.

## Sources of Funding

This study was funded by a donation from Mr Fredrik Lundberg.

## Supporting information

Tables S1–S5
Figures S1–S8
