# Supplementary material for: Effect of Bicuspid Versus Tricuspid Aortic Valve Morphology on the Fate of the Ascending Aorta
Source: J Am Heart Assoc. 2025 Apr 10;14(8):e038013. doi: 10.1161/JAHA.124.038013 (PMC12132860; doi:10.1161/JAHA.124.038013)

SUPPLEMENTAL MATERIAL

Table S1. Distribution of concomitant surgeries among 566 patients operated at Karolinska University Hospital between 2007 and 2013 and included in the ASAP study classified according to tricuspid or bicuspid native aortic valve morphology.

Valve morphology	Aortic valve replacement	Ascending aortic surgery	Root replacement	Aortic valve repair	No.	(%)
TAV	✅	❌	❌	❌	176	(74%)
	✅	✅	❌	❌	7	(3%)
	✅	✅	✅	❌	14	(6%)
	❌	❌	❌	✅	6	(3%)
	❌	✅	❌	❌	22	(9%)
	❌	✅	✅	❌	12	(5%)
	❌	✅	✅	✅	2	(<1%)
BAV	✅	❌	❌	❌	188	(57%)
	✅	✅	❌	❌	52	(16%)
	✅	✅	✅	❌	50	(15%)
	❌	❌	❌	✅	15	(5%)
	❌	✅	❌	❌	14	(4%)
	❌	✅	❌	✅	1	(<1%)
	❌	✅	✅	❌	6	(2%)
	❌	✅	✅	✅	1	(<1%)

TAV = tricuspid aortic valve, BAV = bicuspid aortic valve, ASAP = Advanced Study of Aortic Pathology

Table S2. Baseline characteristics on 206 patients in the ascending aortic diameter cohort who underwent aortic valve or ascending aortic valve surgery at Karolinska University Hospital between January 2007 and March 2013 according to aortic valve morphology

Valve type	Overall	TAV	BAV	SMD	Missing
No.	204	66	138		
Age (mean (SD))	61.1 (11.8)	68.7 (8.7)	57.5 (11.4)	1.096	0.0
Female sex (%)	63 (30.9)	20 (30.3)	43 (31.2)	0.019	0.0
Body mass index (%)				0.207	3.4
<18.5	2 (1.0)	0 (0.0)	2 (1.5)		
18.5-24.9	61 (31.0)	18 (28.6)	43 (32.1)		
25-29.9	87 (44.2)	28 (44.4)	59 (44.0)		
>30	47 (23.9)	17 (27.0)	30 (22.4)		
Smoker (%)				0.398	0.0
Current smoker	19 (9.3)	2 (3.0)	17 (12.3)		
Previous smoker	59 (28.9)	24 (36.4)	35 (25.4)		
Never smoked	126 (61.8)	40 (60.6)	86 (62.3)		
Prior atrial fibrillation (%)	13 (6.4)	6 (9.1)	7 (5.1)	0.157	0.0
Prior myocardial infarction (%)	8 (4.0)	4 (6.1)	4 (2.9)	0.151	1.0
History of cancer (%)	25 (12.3)	10 (15.2)	15 (10.9)	0.128	0.0
COPD or asthma (%)	15 (7.4)	5 (7.6)	10 (7.2)	0.013	0.0
Diabetes mellitus (%)				0.314	0.0
Type 1	2 (1.0)	0 (0.0)	2 (1.4)		
Type 2	22 (10.8)	11 (16.7)	11 (8.0)		
Not diabetic	180 (88.2)	55 (83.3)	125 (90.6)		
Hyperlipidemia (%)	40 (19.6)	15 (22.7)	25 (18.1)	0.115	0.0
Hypertension (%)	103 (51.2)	39 (60.0)	64 (47.1)	0.262	1.5
Prior stroke or TIA (%)	12 (5.9)	6 (9.1)	6 (4.4)	0.187	1.0
Prior lung emboli or DVT (%)	7 (3.5)	3 (4.5)	4 (2.9)	0.085	1.0
Previous inguinal hernia (%)	20 (9.8)	4 (6.1)	16 (11.6)	0.196	0.0

Perioperative ascending aortic diameter (mean (SD))	34.6 (5.1)	32.4 (4.5)	35.6 (5.1)	0.662	0.0
Valvular reconstructive surgery (%)	15 (7.4)	2 (3.0)	13 (9.4)	0.267	0.0
Aortic valve replacement surgery (%)	189 (92.6)	64 (97.0)	125 (90.6)	0.267	0.0
Valve prosthesis type (%)				0.538	0.0
Bioprosthetic aortic valve	124 (60.8)	51 (77.3)	73 (52.9)		
Mechanical valve prosthesis	65 (31.9)	13 (19.7)	52 (37.7)		
No valve replacement	15 (7.4)	2 (3.0)	13 (9.4)		
Valve size, mm (%)				0.585	0.5
22-24	78 (38.4)	19 (29.2)	59 (42.8)		
18-21	59 (29.1)	30 (46.2)	29 (21.0)		
>24	51 (25.1)	14 (21.5)	37 (26.8)		
No valve replacement	15 (7.4)	2 (3.1)	13 (9.4)		

Numbers are n (%) unless otherwise noted. TAV = tricuspid aortic valve, BAV = bicuspid aortic valve, SMD = standardized mean differences, SD = standard deviation, COPD = chronic obstructive pulmonary disease, TIA = transient ischemic attack, DVT = deep vein thrombosis

Table S3. Distribution of aortic event types per tricuspid and bicuspid native aortic valve morphology

Aortic event type	No.	(%)
TAV		
No event	218	(91%)
Aneurysm	4	(2%)
Aneurysm surgery	2	(<1%)
Aneurysm endovascular treatment	4	(2%)
Aortic death	1	(<1%)
Pseudoaneurysm surgery	2	(<1%)
Aortic rupture	2	(<1%)
Type A dissection	3	(1%)
Type B dissection	2	(<1%)
Thoracoabdominal surgery	1	(<1%)
BAV		
No event	314	(96%)
Aneurysm	1	(<1%)
Aneurysm surgery	6	(2%)
Aneurysm endovascular treatment	3	(<1%)
Pseudoaneurysm surgery	1	(<1%)
Aortic rupture	1	(<1%)
Type A dissection	1	(<1%)

Aneurysm = identified but not yet treated aneurysm

Table S4. Distribution of aortic event location according to tricuspid and bicuspid native aortic valve morphology

Aortic event location	No.	(%)
TAV		
Aortic related death	1	(5%)
Ascending aorta	4	(19%)
Above graft	6	(29%)
Descending aorta	10	(48%)
BAV		
Ascending aorta	7	(54%)
Above graft	1	(8%)
Descending aorta	5	(38%)

Above graft = in the remaining ascending aorta beyond the grafted segment

Table S5. Baseline characteristics of 566 patients who underwent aortic valve or ascending aortic valve surgery at Karolinska University Hospital between January 2007 and March 2013 according to adverse aortic event

Valve type	Overall	Aortic event	No aortic event	SMD	Missing
No.	566	34	532		
BAV (%)	327 (57.8)	13 (38.2)	314 (59.0)	0.425	0.0
Age (mean (SD))	63.9 (12.3)	65.0 (10.1)	63.8 (12.5)	0.104	0.0
Female sex (%)	183 (32.3)	11 (32.4)	172 (32.3)	<0.001	0.0
Body mass index (%)				0.224	3.7
<18.5	3 (0.6)	0 (0.0)	3 (0.6)		
18.5-24.9	188 (34.5)	10 (29.4)	178 (34.8)		
25-29.9	222 (40.7)	13 (38.2)	209 (40.9)		
>30	132 (24.2)	11 (32.4)	121 (23.7)		
Smoker (%)				0.340	0.0
Current smoker	48 (8.5)	5 (14.7)	43 (8.1)		
Previous smoker	166 (29.3)	13 (38.2)	153 (28.8)		
Never smoked	352 (62.2)	16 (47.1)	336 (63.2)		
Prior atrial fibrillation (%)	68 (12.0)	1 (2.9)	67 (12.6)	0.367	0.0
Prior myocardial infarction (%)	30 (5.3)	0 (0.0)	30 (5.6)	0.346	0.4
History of cancer (%)	79 (14.0)	6 (17.6)	73 (13.7)	0.108	0.0
COPD or asthma (%)	46 (8.1)	3 (8.8)	43 (8.1)	0.027	0.0
Diabetes mellitus (%)				0.219	0.0
Type 1	5 (0.9)	1 (2.9)	4 (0.8)		
Type 2	55 (9.7)	2 (5.9)	53 (10.0)		
Not diabetic	506 (89.4)	31 (91.2)	475 (89.3)		
Hyperlipidemia (%)	94 (16.6)	4 (11.8)	90 (16.9)	0.147	0.0
Hypertension (%)	297 (52.8)	19 (57.6)	278 (52.5)	0.103	0.5
Prior stroke or TIA (%)	54 (9.6)	2 (6.1)	52 (9.8)	0.139	0.5
Prior lung emboli or DVT (%)	28 (5.0)	1 (3.0)	27 (5.1)	0.104	0.4

Previous inguinal hernia (%)	48 (8.5)	3 (8.8)	45 (8.5)	0.013	0.0
Ascending aortic surgery (%)	181 (32.0)	17 (50.0)	164 (30.8)	0.398	0.0
Aortic root surgery (%)	85 (15.0)	9 (26.5)	76 (14.3)	0.306	0.0
Valvular reconstructive surgery (%)	25 (4.4)	4 (11.8)	21 (3.9)	0.294	0.0
Aortic valve replacement surgery (%)	487 (86.0)	21 (61.8)	466 (87.6)	0.622	0.0
Valve prosthesis type (%)				0.790	0.7
Bioprosthetic aortic valve	337 (60.0)	19 (55.9)	318 (60.2)		
Mechanical valve prosthesis	146 (26.0)	2 (5.9)	144 (27.3)		
No valve replacement	79 (14.1)	13 (38.2)	66 (12.5)		
Valve size, mm (%)				0.700	0.5
18-21	148 (26.3)	4 (11.8)	144 (27.2)		
22-24	178 (31.6)	7 (20.6)	171 (32.3)		
>24	158 (28.1)	10 (29.4)	148 (28.0)		
No valve replacement	79 (14.0)	13 (38.2)	66 (12.5)		

Numbers are n (%) unless otherwise noted. BAV = bicuspid aortic valve, SMD = standardized mean differences, SD = standard deviation, COPD = chronic obstructive pulmonary disease, TIA = transient ischemic attack, DVT = deep vein thrombosis

Figure S1. Absolute (* = standardized) mean differences before and after weighting using optimization -based weights.

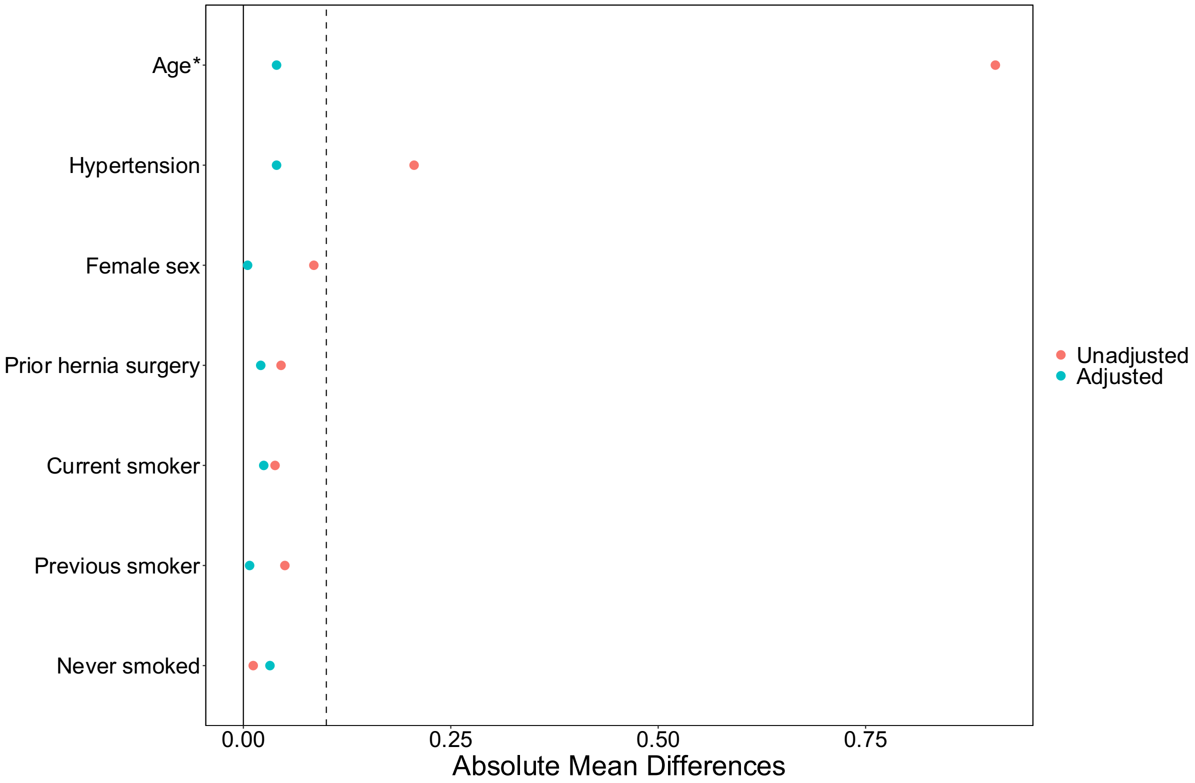

Figure S2. Ascending aortic growth according to patient sex.

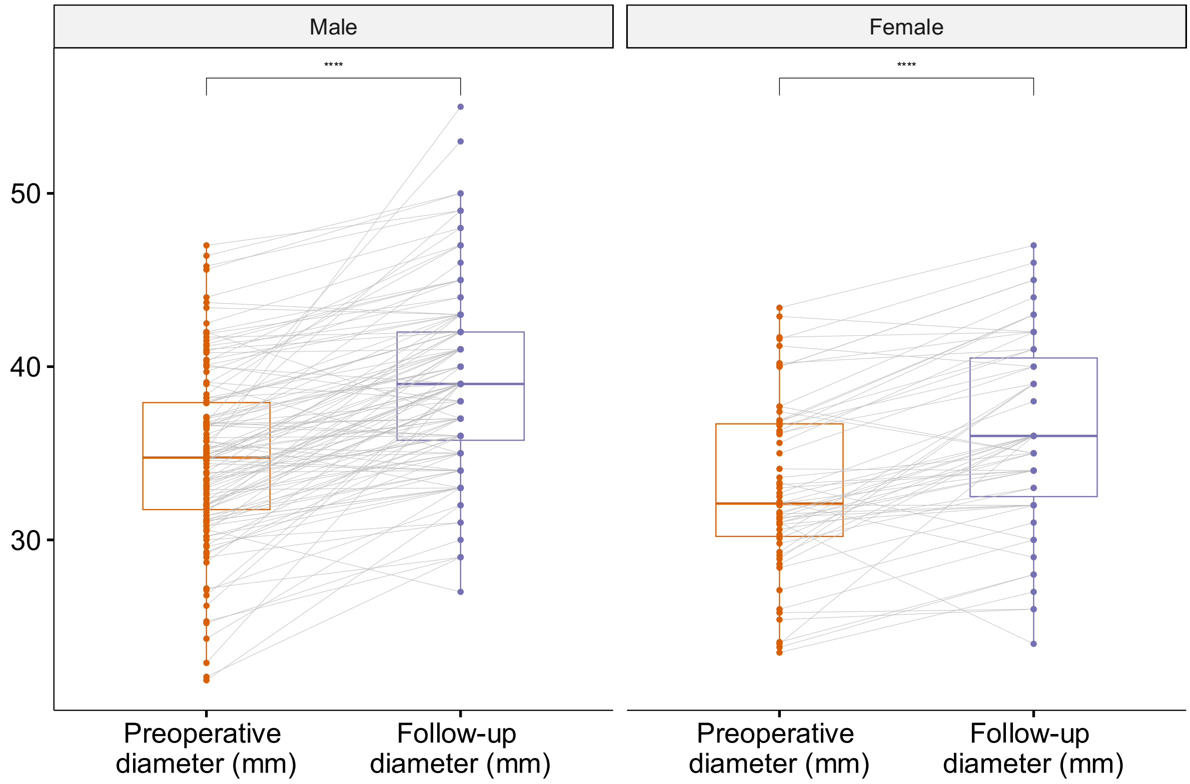

Figure S3. Ascending aortic diameter difference according to native aortic valve morphology and patient sex

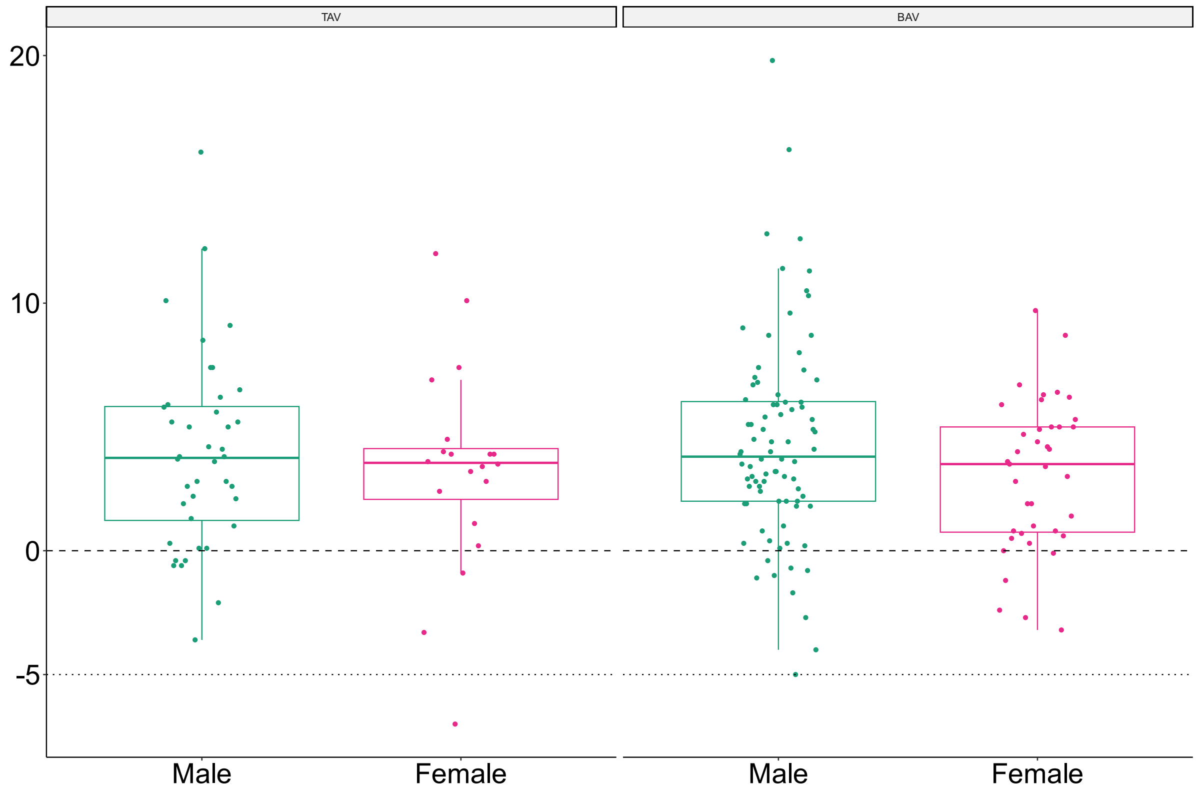

Figure S4. Aortic diameter difference according to native aortic valve morphology and valve pathology. BAV = bicuspid aortic valve, TAV = tricuspid aortic valve, AI = aortic insufficiency, AS = aortic stenosis

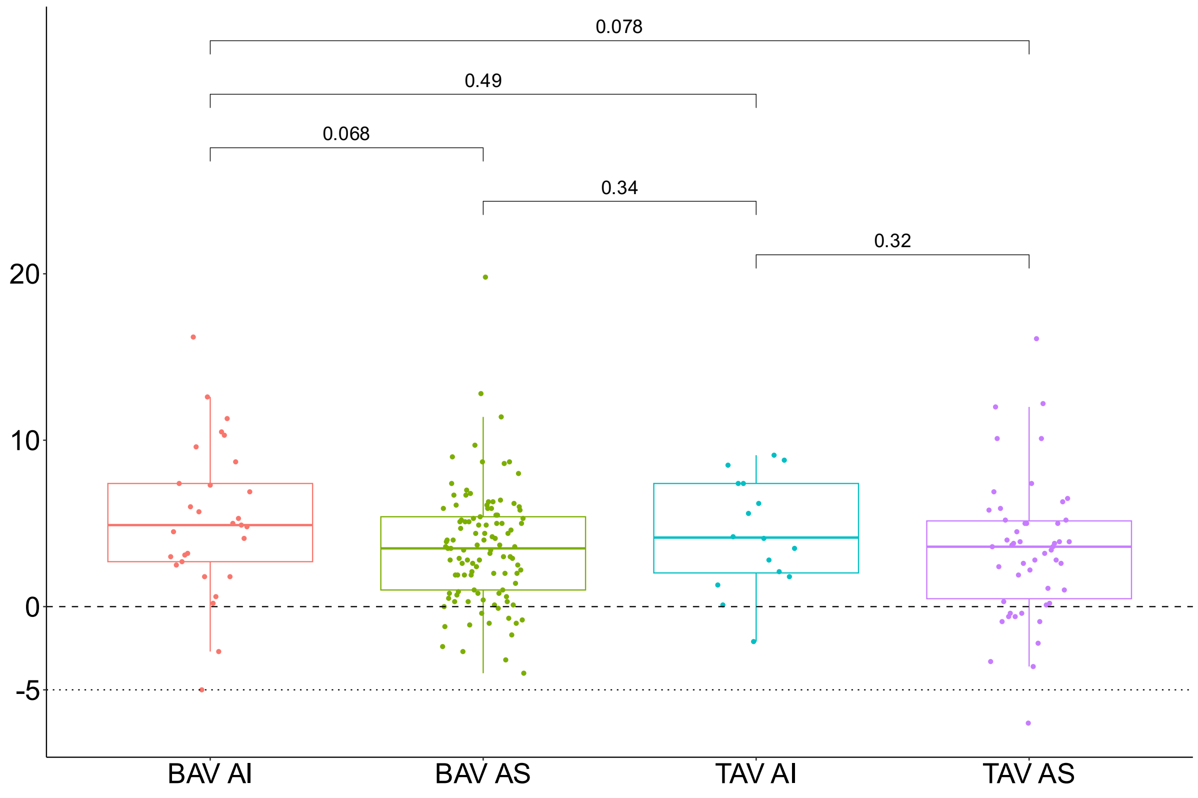

Figure S5. Aalen-Johansen estimated crude cumulative incidence of aortic event among TAV patients with or without concomitant ascending aortic surgery. TAV = bicuspid aortic valve.

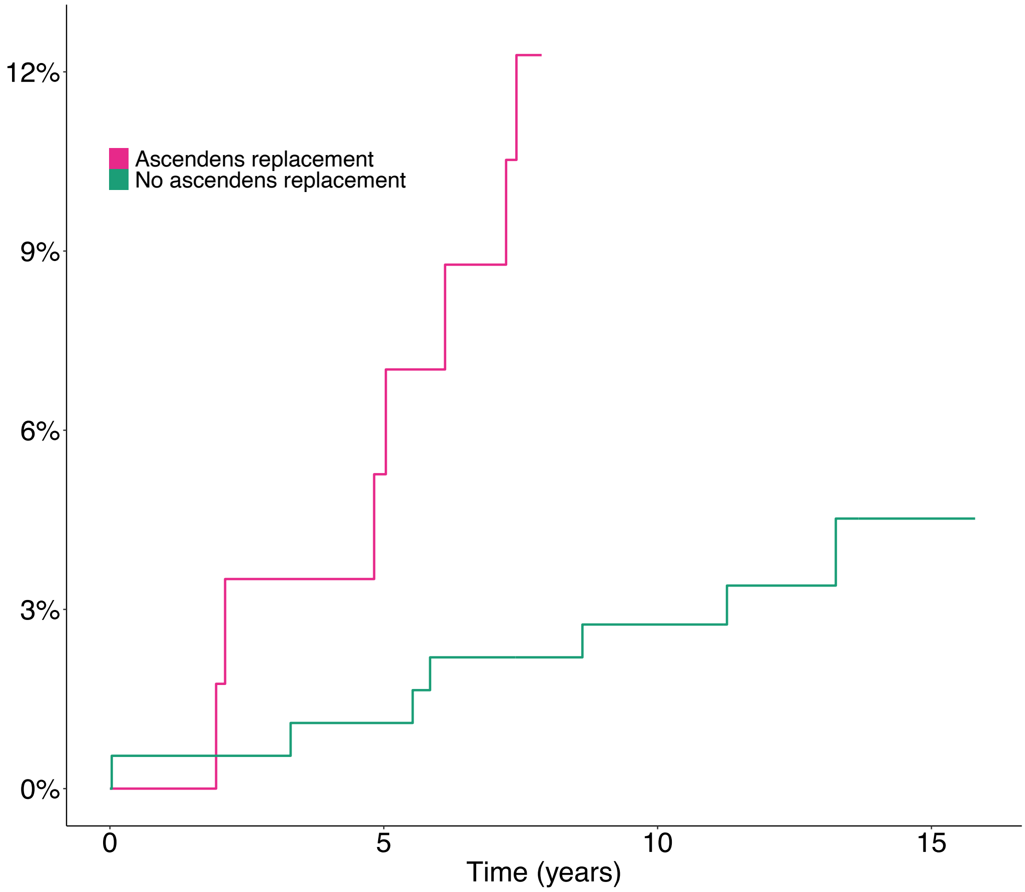

**Ascendens replacement**				
Number at risk	57	49	37	7
Number of events	0	3	8	3
**No ascendens replacement**				
Number at risk	182	161	103	11
Number of events	0	2	3	2

Figure S6. Aalen-Johansen estimated crude cumulative incidence of aortic event among BAV patients with or without concomitant ascending aortic surgery. BAV = bicuspid aortic valve.

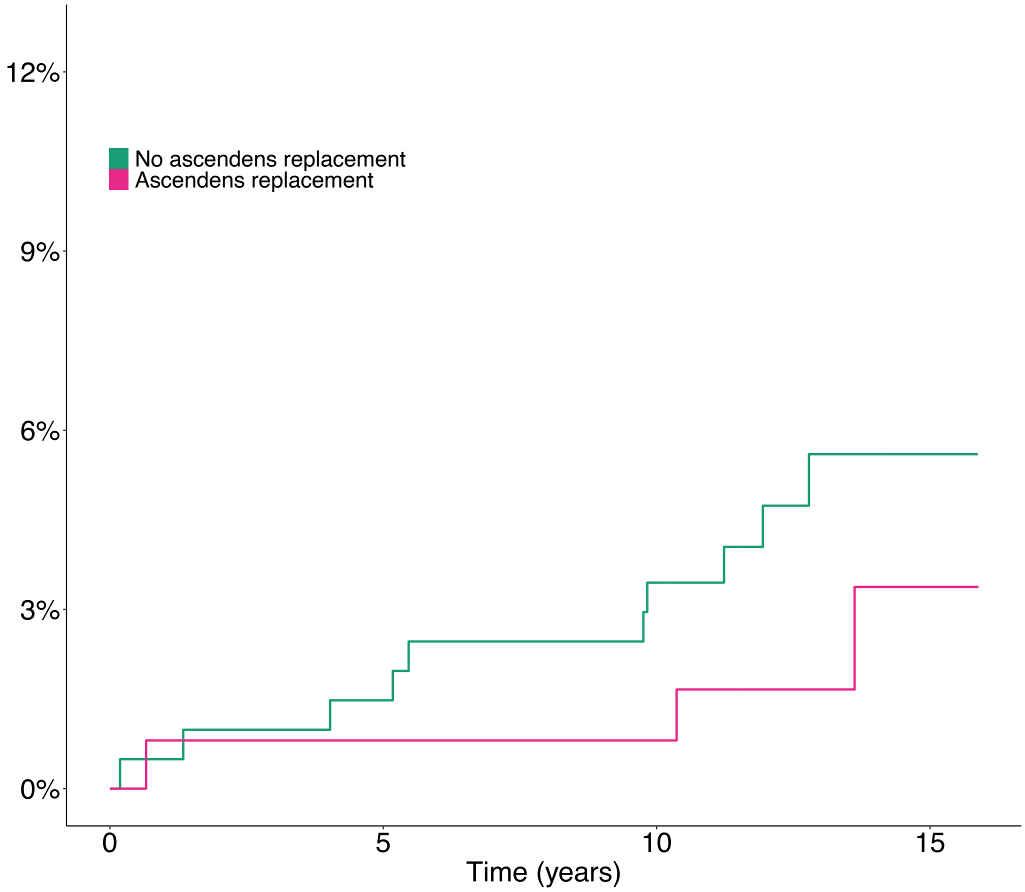

No ascendens replacement

Number at risk	203	186	159	16
Number of events	0	3	4	3

Ascendens replacement

Number at risk	124	118	109	14
Number of events	0	1	0	2

Figure S7. Forest plot of subgroups for all-cause mortality. Constructed using an age-adjusted Cox model. Significance tested using an interaction term of the subgroup and valve phenotype. BAV = bicuspid aortic valve, TAV = tricuspid aortic valve, HR = hazard ratio, CI = confidence interval.

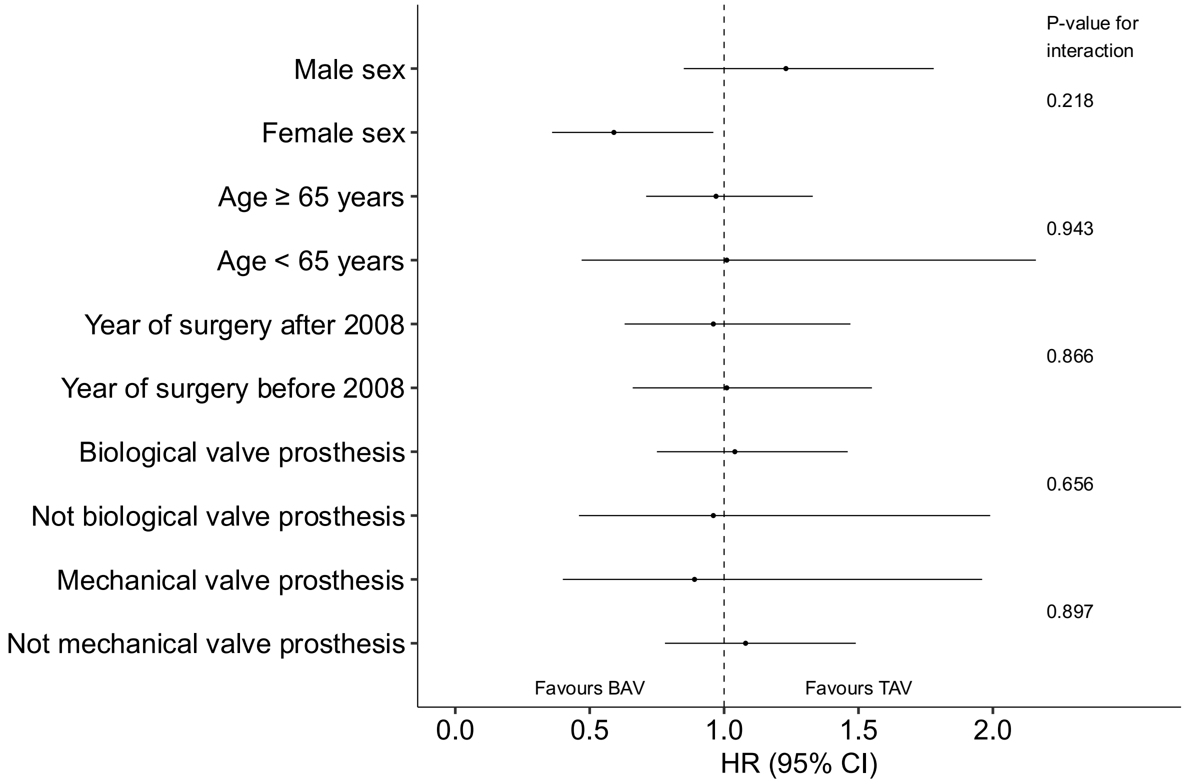

Figure S8. Forest plot of subgroups for aortic events. Constructed using an age-adjusted Cox model. Significa nce tested using an interaction term of the subgroup and valve phenotype. BAV = bicuspid aortic valve, TAV = tricuspid aortic valve, HR = hazard ratio, CI = confidence interval.

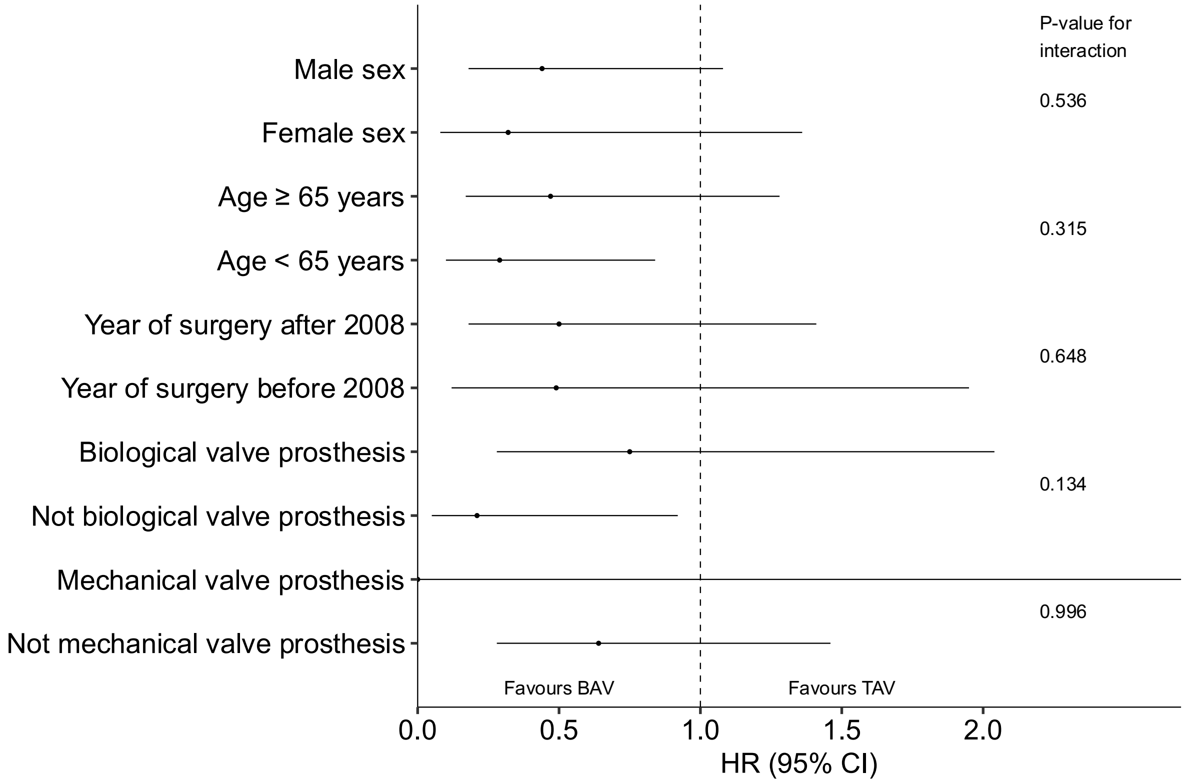

Supplement: Supplementary file 1 — Tables S1–S5 Figures S1–S8 [file JAH3-14-e038013-s001.pdf]
